# Identification of Novel Vaccine Candidates against Multidrug-Resistant *Acinetobacter baumannii*

**DOI:** 10.1371/journal.pone.0077631

**Published:** 2013-10-08

**Authors:** Danilo G. Moriel, Scott A. Beatson, Daniël J. Wurpel, Jeffrey Lipman, Graeme R. Nimmo, David L. Paterson, Mark A. Schembri

**Affiliations:** 1 Australian Infectious Diseases Research Centre, the University of Queensland, Brisbane, Queensland, Australia; 2 School of Chemistry and Molecular Biosciences, the University of Queensland, Brisbane, Queensland, Australia; 3 Department of Intensive Care Medicine, Royal Brisbane and Women’s Hospital, and Burns, Trauma and Critical Care Research Centre, University of Queensland, Brisbane, Queensland, Australia; 4 Pathology Queensland Central Laboratory, Brisbane, Queensland, Australia; 5 University of Queensland Centre for Clinical Research, Royal Brisbane and Women’s Hospital Campus, University of Queensland, Brisbane, Queensland, Australia; Monash University, Australia

## Abstract

*Acinetobacter baumannii* is an emerging opportunistic bacterium associated with nosocomial infections in intensive care units. The alarming increase in infections caused by *A. baumannii* is strongly associated with enhanced resistance to antibiotics, in particular carbapenems. This, together with the lack of a licensed vaccine, has translated into significant economic, logistic and health impacts to health care facilities. In this study, we combined reverse vaccinology and proteomics to identify surface-exposed and secreted antigens from *A. baumannii*. Using *in silico* prediction tools and comparative genome analysis in combination with *in vitro* proteomic approaches, we identified 42 antigens that could be used as potential vaccine targets. Considering the paucity of effective antibiotics available to treat multidrug-resistant *A. baumannii* infections, these vaccine targets may serve as a framework for the development of a broadly protective multi-component vaccine, an outcome that would have a major impact on the burden of *A. baumannii* infections in intensive care units across the globe.

## Introduction

*Acinetobacter baumannii* is an emerging bacterial pathogen and a leading cause of endemic and epidemic nosocomial infections in hospitals, in particular in intensive care units. Infections caused by *A. baumannii* include ventilator-associated pneumonia, bloodstream infections, secondary meningitis, wound, skin, soft-tissue and urinary tract infections [1]. The rapid emergence of multidrug-resistant (MDR) *A. baumannii* strains has left limited treatment options for patients, with most strains resistant to clinically useful antibiotics such as aminoglycosides, fluoroquinolones, beta-lactams (including carbapenems), tetracyclines and trimethoprim-sulfamethoxazole [2].

Despite its increased propensity to acquire resistance to antibiotics, *A. baumannii* is typically clonal and three clinically-associated international MDR *A. baumannii* clones (originally named European Clones [EC] I, II and III) were first described [3]. More recently, epidemiological studies employing multi-locus sequence typing (MLST) have defined several dominant clonal complexes (CC) that correlate with the previously identified European clone scheme, namely CC1 (EC I), CC2 (EC II) and CC3 (EC III) and further analysis of the population structure of *A. baumannii* clinical isolates has confirmed its limited genetic diversity [4,5].

The paucity of antibiotics that remain active against MDR *A. baumannii* indicates a possible role for vaccination as an alternative strategy to effectively reduce the burden and impact of infections caused by this pathogen. Protection against *A. baumannii* infections has already been achieved by active and passive immunization in mice using OmpA [6], Ata [7] and Bap [8], indicating the potential and feasibility of vaccination. However, the solubility, variability and prevalence of these antigens represent significant obstacles for the delivery of a broadly protective vaccine.

Several vaccinology strategies have already been used for the identification of protective vaccine candidates against emerging and concerning human pathogens. Reverse vaccinology is an *in silico* approach that involves the mining of genome sequences using comparative analysis and prediction tools for the identification of antigens predicted to be highly prevalent, soluble and surface-exposed/secreted. Another vaccinology approach involves the proteomic analysis of outer membrane vesicles (OMVs) released by bacteria, which provides deep insight into the proteins that could be exposed on the bacterial surface and potentially accessible to antibodies. Both technologies are frequently used as standalone strategies, and have contributed significantly to the identification of novel protective antigens. For example, vaccine antigens that provide protection against extraintestinal pathogenic *Escherichia coli* [9,10] and *Neisseria meningitidis* serogroup B [11,12] have been identified using these methods.

Here, we employed a combinatorial approach encompassing both reverse vaccinology and proteomics to identify vaccine antigens of *A. baumannii*. Overall, our approach identified multiple novel candidate antigens that should form a framework for future vaccine development.

## Results

### Selection of *A. baumannii* genome sequences

**Figure 1:**
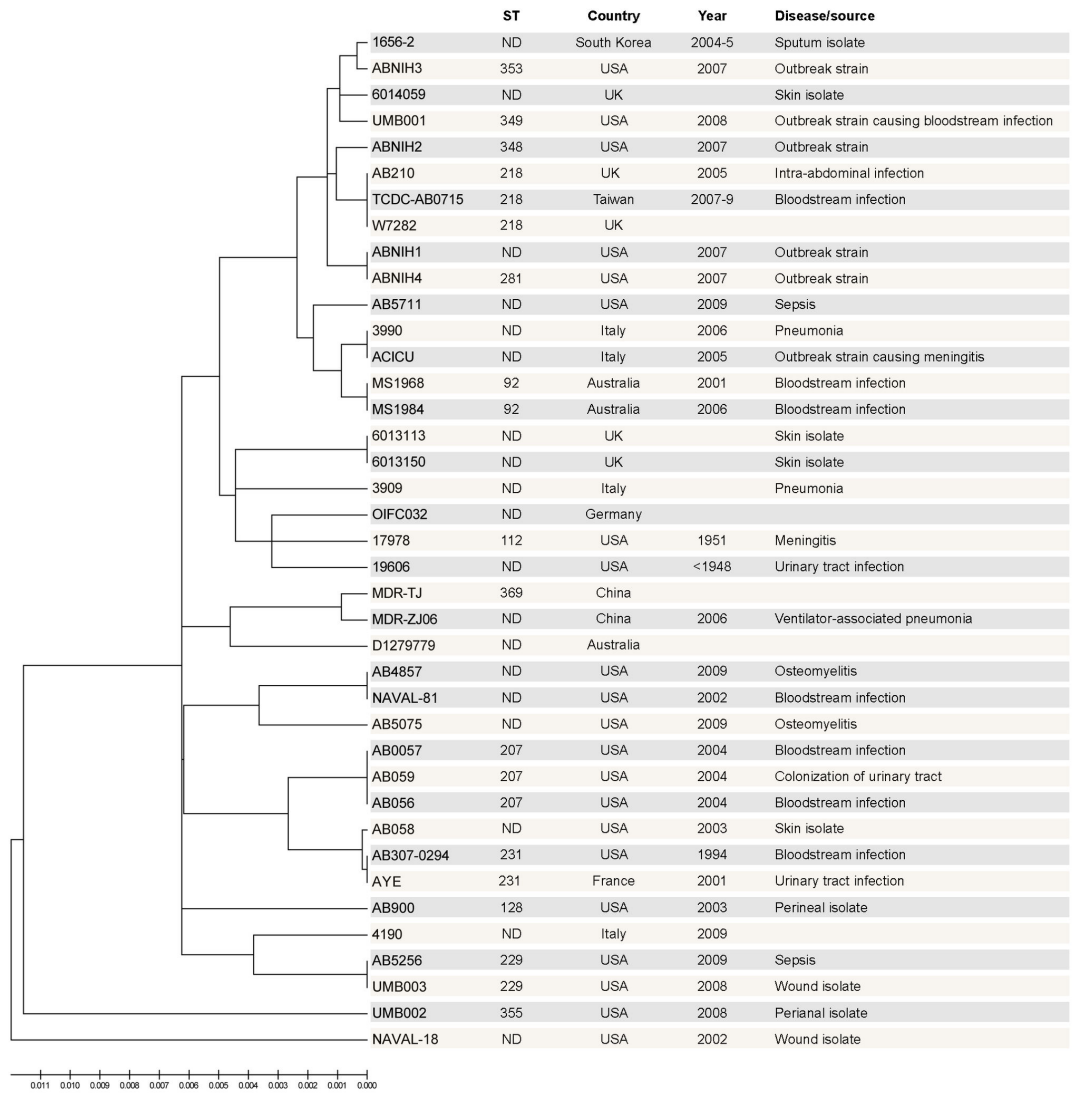
Collection of *A. baumannii* genome sequences used in this study. The evolutionary history was inferred using 39 genome sequences, since only partial sequence data was available for some of the strains analyzed (W6976, SDF, A118, WM99c). ND, not defined in the PubMLST database.

In order to ensure broad coverage of the *A. baumannii* species in our analyses, we used all of the available genome sequences from public databases, which included 10 complete and 31 draft genomes (Table S1). Few redundancies were observed in this dataset, since most of the strains differed with respect to MLST, year of isolation, geographic location, associated disease and source of isolation (Figure 1). The only exceptions were strains 6013113 and 6013150, AB0057 and AB056, and MS1968 and MS1984; the latter two strains represent draft genome sequences of emerging ST92 strains isolated from an intensive care unit in Australia during outbreaks in 2001 and 2006, respectively.

### *In silico* identification of potential vaccine candidates

At the time of initiation of this study, the complete genome sequence of ten *A. baumannii* strains was available on the NCBI database (Table S1). These strains were used to perform *in silico* reverse vaccinology analysis to identify novel vaccine candidate antigens. Where possible, the annotated genome sequence of the MDR *A. baumannii* strain AYE was used as a reference. *A. baumannii* AYE was isolated during a nationwide outbreak in France and was the first *A. baumannii* strain from which an extended-spectrum beta-lactamase VEB-1 was identified [13]. The subcellular localization of proteins was predicted for the ten sequenced strains, and all proteins >100 amino acids containing a predicted signal sequence and with a PSORTb prediction of outer membrane, extracellular or unknown, were selected. Since *A. baumannii* is an emerging and poorly characterized pathogen, clearly illustrated by the high number of hypothetical proteins in the annotated genomes, we tried to establish their correlation to mechanisms of host-pathogen interaction through the presence of relevant domains (Pfam) as well as their sequence (PSI-Blast) and structural (PHYRE2) similarity to known virulence factors. This led to the identification of 234 non-redundant antigen candidates from the ten different *A. baumannii* genome sequences (Table S2).

### *In vitro* identification of outer membrane and secreted proteins

OMVs are spherical nanovesicles secreted by many Gram-negative bacteria and composed of lipopolysaccharides, proteins, lipids, and DNA or RNA [14]. *A. baumannii* secrete OMVs during *in vitro* growth as a vehicle for the delivery of effectors to host cells [15]. We exploited this property to identify surface-exposed antigens (i.e. potential vaccine targets) from *A. baumannii* AYE and the ST92 strains. As a parallel approach, we also evaluated the secretome of these strains. These analyses led to the identification of 122 *A. baumannii* proteins present in the secretome and/or in EDTA-heat induced OMVs (Table S3) that had a PSORTb prediction of outer membrane, extracellular or unknown and a potential signal sequence. Proteins with predicted cytoplasmic, cytoplasmic membrane or periplasmic origins, and whose subcellular localization was supported as either cytoplasmic or periplasmic by comparative structural and sequence analysis, were considered as contaminants of the *in vitro* preparations and not assessed further.

### Selection of the best vaccine candidates

**Figure 2:**
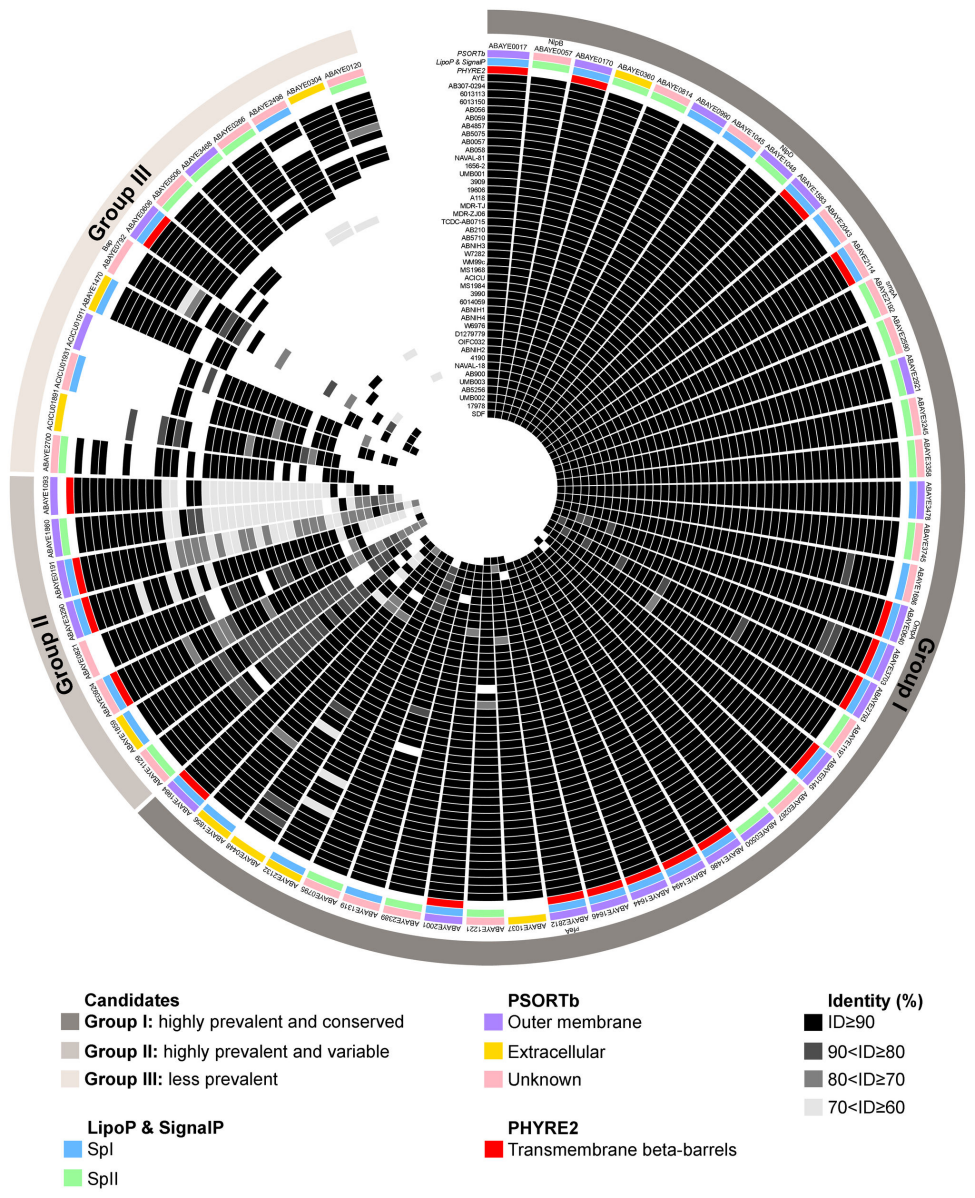
Selection of potential vaccine candidates against *A. baumannii*. The 62 antigens overlapping both reverse vaccinology and proteomic analysis are represented, including their prevalence in the 43 strains used in this study. Predictions of subcellular localization (PSORTb) and signal sequence (LipoP and SignalP) are indicated. Potentially insoluble antigens due to transmembrane beta-barrel structure prediction (PHYRE2) are indicated. Strains and antigens are sorted according to prevalence, incidence and sequence variability.

The list of *A. baumannii* surface-exposed or secreted antigens was refined by comparing data from the *in silico* reverse genetic analysis and the *in vitro* proteomic analysis. This led to a list of 62 antigens that overlapped from both approaches (Figure 2). The prevalence and variability of genes encoding each of the 62 antigens was determined by *in silico* analysis of available complete (n=10) and draft (n=33) *A. baumannii* genomes. This analysis revealed the presence of three distinct groups: highly prevalent and conserved antigens (Group I), which share a sequence identity above 90% in more than 70% of the collection; highly prevalent and variable antigens (Group II), which share a sequence identity below 90% (but >60%) in more than 70% of the collection; and low prevalence antigens (Group III), present in less than 70% of the strains (Figure 2). All of the strains examined except SDF possessed more than 70% of the vaccine candidates; *A. baumannii* strain SDF was isolated from a human body louse and is sensitive to most antibiotics. Each antigen was also assessed for potential solubility by predicting structural conformation using PHYRE2. This analysis revealed that 20 antigens contain a predicted beta-barrel structure (Table S4) and thus are likely to be insoluble upon overexpression, a major obstacle for vaccine development.

Taken together, our combined analyses identified 42 top vaccine targets based on cellular location, prevalence, sequence variability and solubility (Table 1). These candidate proteins were predicted to be soluble and, despite the low prevalence of some proteins (Group III), they could cover all the strains analyzed using a multi-component vaccine. Grouping according to their predicted function/domains revealed the following distribution: outer membrane lipoproteins (n=18); adhesins and haemagglutinins (n=10); enzymes and toxins (n=9); solenoid repeat proteins (n=2); and hypothetical proteins (n=3). 

**Table 1:** *A. baumannii* top vaccine candidates.

**Locus tag**	**S^a^**	**OMV**	**Product**	**Length^b^**	**PSORTb**	**SP^c^**	**Pfam/Potential function**
*Outer membrane lipoproteins*							
ABAYE0057	-	+	NlpB lipoprotein	202	Unknown	SpII	NlpB/DapX lipoprotein
ABAYE0120	-	+	Hypothetical protein	153	Unknown	SpII	
ABAYE0266	-	+	Lipoprotein	198	Unknown	SpII	
ABAYE0267	-	+	Hypothetical protein	233	Unknown	SpII	
ABAYE0360	-	+	Hypothetical protein	144	EC	SpII	
ABAYE0500	-	+	Lipoprotein	160	OM	SpII	OmpA (PF00691)
ABAYE0506	-	+	Hypothetical protein	402	Unknown	SpII	
ABAYE0814	-	+	Hypothetical protein	141	Unknown	SpII	
ABAYE1048	-	+	Lipoprotein	277	OM	SpII	LysM domain (PF01476)
ABAYE1221	+	+	Hypothetical protein	127	Unknown	SpII	
ABAYE1860	+	-	Hypothetical protein	326	OM	SpII	
ABAYE2498	-	+	Hypothetical protein	197	Unknown	SpI	
ABAYE2700	-	+	Hypothetical protein	230	Unknown	SpII	
ABAYE2921	-	+	Lipoprotein	133	OM	SpII	SmpA/OmlA family (PF04355)
ABAYE3245	-	+	Lipoprotein	170	Unknown	SpII	LPS-assembly (PF04390)
ABAYE3358	-	+	Hypothetical protein	197	Unknown	SpII	
ABAYE3468	+	+	Hypothetical protein	334	OM	SpII	
ABAYE3745	-	+	Hypothetical protein	138	Unknown	SpII	
*Adhesins and haemagglutinins*							
ABAYE0304	+	+	Fimbrial protein	158	EC	-	Adhesion (PF13544)
ABAYE0448	+	+	Hypothetical protein	808	EC	-	Haemagglutinin-like
ABAYE0792	+	+	Hypothetical protein	8201	Unknown	-	Adhesion (PF13754)
ABAYE0821	+	+	Hypothetical protein	3370	Unknown	-	Adhesion (PF13754)
ABAYE1037	+	+	Hypothetical protein	729	EC	-	Adhesion (PF13754)
ABAYE1319	+	-	CsuA/B; fimbrial protein	181	Unknown	SpI	Adhesion (PF05229)
ABAYE1470	+	-	Biofilm protein	177	EC	SpI	Adhesion (PF05229)
ABAYE1856	+	+	Fimbrial protein	178	EC	SpI	Adhesion (PF00419)
ABAYE1859	+	+	Fimbrial adhesin	337	EC	SpI	Adhesion (PF00419)
ABAYE2132	+	-	Fimbrial protein	210	EC	SpI	Adhesion (PF00419)
*Enzymes and toxins*							
ABAYE0795	-	+	Metalloprotease	246	Unknown	SpII	Metalloprotease-like
ABAYE0990	-	+	Protease	921	OM	SpI	Metalloprotease (PF05193)
ABAYE1129	+	+	Hydrolase	486	Unknown	SpII	Alpha/beta hydrolase (PF12697)
ABAYE2043	-	+	Metallopeptidase	678	Unknown	SpI	Metalloprotease M3 (PF01432)
ABAYE2389	+	+	Hypothetical protein	145	Unknown	SpII	DUF (PF03891), hemolysin-like
ABAYE2590	-	+	Hypothetical protein	260	Unknown	SpII	Metalloprotease M48 (PF01435)
ACICU_01891	+	-	RTX toxin	1451	EC	-	Hemolysin Ca^2+^-binding (PF00353)
ACICU_01911	+	-	Exoprotein	2142	OM	-	MafB19-like deaminase (PF14437)
ACICU_01931	-	+	Esterase/lipase	337	Unknown	SpI	Alpha/beta hydrolase fold (PF07859)
*Solenoide repeat proteins*							
ABAYE1197	-	+	Hypothetical protein	184	Unknown	SpII	Tetratrico peptide repeats
ABAYE2192	-	+	Hypothetical protein	314	Unknown	SpII	Tetratrico peptide repeats
*Hypothetical proteins*							
ABAYE1045	-	+	Hypothetical protein	141	Unknown	SpI	
ABAYE1686	-	+	Hypothetical protein	163	Unknown	SpI	
ABAYE3478	-	+	Hypothetical protein	380	OM	SpI	

^a^ Secretome; ^b^ amino acids; ^c^ Signal peptide

Abbreviations: LPS: lipopolysaccharide; DUF: domain of unknown function; OM: outer membrane; EC: extracellular.

## Discussion

*A. baumannii* is an emerging MDR pathogen frequently isolated from clinical settings. The healthcare and economic impact of *A. baumannii* infections, particularly in intensive care units, highlight the urgent need to develop new approaches to treat and prevent such infections. Here, we have defined a novel group of *A. baumannii* candidate vaccine antigens based on their predicted subcellular location, prevalence, sequence conservation and predicted solubility.

The resistance of *A. baumannii* to multiple antibiotics occurs through intrinsic mechanisms, and through the gain of laterally acquired resistance genes [16]. Given its propensity to rapidly and efficiently develop resistance, vaccination represents a viable alternative strategy to prevent infections caused by *A. baumannii*. In fact, several conventional vaccinology approaches have been used for the identification of potential vaccine targets. Whole-cell [17], OMV [18] and outer membrane preparations [19] have been shown to confer active and passive protection in a murine model of disseminated sepsis against different strains. These approaches have the advantage of providing responses to several surface-exposed epitopes simultaneously, however endotoxin contamination remains a limiting factor for their development and translation to human use. In contrast, subunit preparations offer a viable alternative for vaccine development. Such vaccines stimulate the production of opsonizing and/or functional antibodies, which target specific components present on the bacterial cell surface or secreted virulence factors that interact with host epithelial cells and cause disease. When soluble, subunit antigens are relatively easy to obtain on a large scale and production processes are highly reproducible, assisting approval from regulatory agencies.

Reverse vaccinology takes advantage of the many genome sequence datasets available in the public domain and represents a targeted approach for the discovery of novel surface antigens. The use of a sequenced based approach ensures the evaluation of all putative proteins encoded within the genome of a given strain, however is limited by the availability of effective search tools to predict protein subcellular location. The accuracy of genome annotation and the quality of training datasets therefore impact the analysis outcome. Conversely, proteomic approaches allow the identification of bacterial surface antigens, but may be limited by sensitivity (i.e. for detection of poorly expressed proteins) or lack of expression of certain antigens under some growth conditions. Thus, methods for bacterial growth, cell fractionation and protein preparation may lead to variable or even inconsistent results.

In this study, we adopted an approach that combines these methods and provides an extra filtering step in our definition of candidate vaccine antigens for MDR *A. baumannii*, and we predict this will be more informative than data obtained from each method employed as a standalone technique for vaccine antigen selection. Ten complete and thirty-one draft *A. baumannii* genomes were used for reverse vaccinology analysis, leading to the identification of 234 putative outer membrane or secreted proteins based on our strict criteria to define protein subcellular localization. The strains employed covered a wide spectrum of geographic locations and disease states, and contained representative strains from the dominant *A. baumannii* clones currently circulating the globe. The proteomic approach involved the analysis of three *A. baumannii* clinical isolates, and resulted in the identification of 122 secreted or OMV-associated proteins. Overall, sixty-two proteins were identified from both approaches. This list was further refined by the removal of twenty proteins predicted to contain a beta-barrel structure (Table S4). Such proteins are generally buried within the outer membrane, and interact with the immune system via external loops presented as conformational epitopes. Many of these eliminated proteins were also predicted to be involved in phenotypes associated with a high level of redundancy, such as iron acquisition and transport functions. For example, BauA (ABAYE1093), is a siderophore receptor involved in the transport of acinetobactin [20] and intracellular survival within epithelial cells [21].

Our final refined list of *A. baumannii* proteins contained 42 putative antigens. This included OmpA (ABAYE0640) and Bap (ABAYE0792), both of which have been examined previously as vaccine candidates against *A. baumannii* [6,8]. OmpA is an outer membrane and secreted lipoprotein that contributes to biofilm formation, interaction with epithelial cells [22] and induction of apoptosis by epithelial cells [23]. Immunization of diabetic mice with OmpA in combination with aluminum hydroxide adjuvant has been shown to induce high anti-OmpA antibody titers and resulted in reduced tissue bacterial burden and improved survival of mice following intravenous infection with *A. baumannii* [6]. Despite this promising level of protection, others have shown OmpA is soluble and active when recovered from the supernatant of bacterial cultures, but insoluble when expressed as a recombinant protein [24]. Vaccination of mice with Bap, a surface-exposed adhesin involved in biofilm formation, has also been performed. Immunization with Bap conferred protection in a murine model of sepsis and led to a significant increase in survival, as well as a reduction in bacterial counts in the liver and spleen of infected mice [8].

Based on sequence and structural analysis, the 42 candidate antigens could be divided into five groups. The major group comprised outer membrane lipoproteins (n=18). Outer membrane lipoproteins have previously been identified as major vaccine targets against other bacterial pathogens [11]. For example, factor H-binding lipoprotein elicits antibodies against *N. meningitidis* serogroup B [25], and constitutes an important component of Bexsero, the first vaccine developed using reverse vaccinology [26]. Ten proteins predicted to be associated with adhesion, including five putative fimbrial proteins and Bap, were also identified. Fimbrial proteins have previously been shown to constitute effective vaccines for some pathogens (e.g. *Bordetella pertussis* [27] and uropathogenic *E. coli* in animal infection models [28,29]). Furthermore, our detection of these proteins using proteomics is consistent with their role in biofilm formation [30,31]. Nine enzymes/toxins were identified in our final list, and given that most MDR *A. baumannii* strains are hemolytic [32], these proteins may also represent important targets for vaccine development. Enzymes/toxins play an important role in the scavenging of nutrients by bacterial pathogens, and many are toxic to the human host. Functional antibodies that block the activity of toxins generally reduce the severity of infection, and several highly effective, licensed vaccines use inactivated toxins (toxoids) to raise protective antibodies (e.g. anthrax, diphtheria, pertussis and tetanus toxins). Two solenoid repeat proteins were identified in our analysis. These proteins often interact with other proteins and include Tetratrico Peptide Repeat proteins, Pentatrico Peptide Repeat proteins and Sel1-like repeat proteins. Characterized examples from this family include the *Helicobacter* cysteine-rich protein (Hcp), and the newly described c5321 protective antigen against extraintestinal pathogenic *E. coli* [9]. Finally, three hypothetical proteins with no sequence or structural homology to any characterized proteins in the NCBI non-redundant protein database were identified.

The prevalence and variability of each of the 42 antigens was also assessed using the ten complete genome sequenced strains as well as an additional 33 strains for which a draft genome sequence was available. The antigens could be classified into three groups based on prevalence and amino acid sequence conservation, with antigens in Group I (n=41) representing the most likely candidates for vaccine antigens. However, antigens in Group II and III include many of the potential adhesins, and thus we cannot rule out their use in a potential multi-component subunit vaccine. We note also that some proteins that offer potential as vaccine candidates may have been missed in our analysis. Such an example is Ata, an autotransporter protein that plays an important role in biofilm formation and binding to extracellular matrix and basal components [33,34]. Vaccination with Ata has been shown to attenuate infection in a pneumonia murine passive model using immunocompetent and immunocompromised mice [7]. Ata (A1S_1032) is present in the ST92 strains and was identified by reverse vaccinology (Table S2), but we were unable to detect its expression *in vitro* by HPLC-MS/MS analysis. Another example is PKF (ABAYE0936), a secreted serine protease that confers resistance to complement mediated killing and biofilm formation [35]. Predicted to be a periplasmic protein by PSORTb, PKF was initially discarded by the genomic approaches but identified by the proteomic analysis (Table S5). Even though our combined approach could miss some potential vaccine antigens, the standalone approaches are complementary and thus provide a backup list of potential targets for future consideration. We note also that a limitation of our approach was the inability to infer the correct orientation of hypothetical outer membrane proteins with respect to the extracellular or periplasmic space by sequence and structural analysis alone. Thus, further analysis of these proteins is required to confirm their suitability as vaccine candidate antigens.

Murine sepsis models using subcutaneous or intramuscular immunization and either intravenous [6] or intraperitoneal [8,17-19] challenge have previously been used to test the efficacy of potential vaccine candidates against *A. baumannii*. The 42 vaccine antigens identified in this study could feasibly be tested in a murine sepsis model to examine their ability to protect against bloodstream infections, one of the most important clinical manifestations of MDR *A. baumannii* [1]. A recent international surveillance study reported the association of *A. baumannii* with 8.8% of ICU infections (ranging from 3.7% to 19.2% according to geographical region) [36]. *A. baumannii* has also emerged as an important cause of infections resulting from injuries sustained by military personnel during recent operations in Iraq and Afghanistan [37]. Therefore, a broadly protective vaccine against *A. baumannii* may have a major impact on some high risk groups, including ICU patients, injured military personal, patients undergoing elective surgery, diabetics and hemodialysis patients.

In conclusion, this study provides the first comprehensive analysis of *A. baumannii* strains for vaccine purposes, and has identified potential antigens that could form a framework for the design of a novel and broadly protective vaccine targeted against MDR *A. baumannii*. Given the rapid emergence and dissemination of MDR *A. baumannii* strains in healthcare settings across the globe, such a vaccine would address an urgent and currently unmet need. Our combined use of reverse vaccinology and proteomics provides an excellent example of the high throughput power of these complementary strategies for the identification of potential vaccine targets when an appropriate collection of genome sequences from epidemiologically relevant strains is available. Future work will now be targeted towards the characterization of the proteins identified in our analyses, as well as their evaluation in animal infection models as vaccine antigens.

## Materials and Methods

### Bacteria and growth conditions

*A. baumannii* strain AYE is a MDR urinary tract infection isolate involved in a nationwide outbreak in France in 2001 [38]. *A. baumannii* ST92 strains (MS1968 and MS1984) are MDR outbreak strains isolated from the Royal Brisbane and Women’s Hospital, Australia [39]. Bacteria were grown in minimal medium supplemented with casamino acids (M9CA) composed of: 0.60% Na_2_HPO_4_, 0.30% KH_2_PO_4_, 0.30% casamino acids, 0.20% glucose, 0.20% thiamine, 0.10% NH_4_Cl, 0.05% NaCl, 1 mM MgSO_4_ and 0.1 mM CaCl_2_. Initial OD was set to 0.050 and incubated at 37°C under shaking (180 rpm) until OD 0.500 was reached. Cells were centrifuged at 10,000*g* for 10 minutes at 4°C. Pellet was used to obtain EDTA-heat induced OMVs and supernatant for secretome analysis.

### EDTA-heat induced OMVs

The formation of OMVs was induced by EDTA as previously described [40]. Briefly, cells were washed 3 times with 1 ml ice-cold PBS and centrifuged at 10,000*g* at 4°C for 5 minutes. Cells were resuspended in 2 ml EDTA buffer (0.15 M NaCl, 0.05 M Na_2_HPO_4_, 0.01 M EDTA, pH 7.4) and incubated at 56°C for 30 minutes. The pellet was separated by centrifugation at 10,000*g* for 10 minutes at 4°C and supernatant was filtered using 0.22 µm low protein binding PES filters (Millipore) and centrifuged at 200,000*g* for 90 minutes at 4°C. OMVs were resuspended in PBS and precipitated overnight at 4°C by adding trichloroacetic acid (TCA) at a final concentration of 10%.

### Secretome analysis

Supernatant was centrifuged twice at 10,000*g* for 20 minutes at 4°C and filtered using 0.22 µm low protein binding PES filters (Millipore). Sample was centrifuged at 200,000*g* for 90 minutes at 4°C and precipitated overnight at 4°C by adding TCA at a final concentration of 10%.

### HPLC-MS/MS proteome analysis

After precipitation, OMVs and secretome preparations were centrifuged at 18,000*g* for 30 minutes at 4°C. Pellet was washed with 1 ml 10% TCA and centrifuged at 18,000*g* for 15 minutes at 4°C. Pellet was resuspended in ice-cold ethanol and centrifuged at same speed. Pellet was dried using a Speed Vac at low drying rate for 20 minutes. Dried pellet was resuspended in 50 µl resuspension buffer (50 mM ammonium bicarbonate, 3 M urea, 5 mM DTT) and 5 µl fresh 250 mM iodoacetamide, 50 mM ammonium bicarbonate was added and incubated for 30 minutes at room temperature in the dark. After incubation, 100 µl 50 mM ammonium bicarbonate was added to reduce the concentration of urea to 1 M. Digestion was performed using 1 µg/µl trypsin overnight at 37°C. Samples were analyzed by HPLC-MS/MS and peptide fingerprint was evaluated using ProteinPilot software 4.0 and AYE protein database.

### Bioinformatic tools

Sequence comparisons were performed using FASTA36 package [41] and PSI-BLAST tools [42]. Subcellular localization was predicted by PSORTb 3.0 [43] and signal sequence was predicted by SignalP 4.0 [44], LipoP 1.0 [45] or manual sequence analysis. Domains were identified using the Pfam database [46]. Structural homologies and predictions were performed using PHYRE2 [47]. Phylogenetic analysis was performed using seven housekeeping genes as previously described [4] and tree was constructed in MEGA5 [48]. MLST was conducted by direct comparison of housekeeping genes to the *A. baumannii* PubMLST database (http://pubmlst.org/abaumannii).

## Supporting Information

Table S1 ***A. baumannii* genome sequences used in this study.**
(DOCX)

Table S2 ***A. baumannii* antigens identified through the reverse vaccinology approach.**
(DOCX)

Table S3 ***A. baumannii* OMV and secretome.**
(DOCX)

Table S4 ***A. baumannii* potentially insoluble proteins.**
(DOCX)

Table S5 ***A. baumannii* periplasmic proteins found in OMV and secretome.**
(DOCX)
