# Supplementary material for: Identification of Novel Vaccine Candidates against Multidrug-Resistant Acinetobacter baumannii
Source: PLoS One. 2013 Oct 8;8(10):e77631. doi: 10.1371/journal.pone.0077631 (PMC3792912; doi:10.1371/journal.pone.0077631)
Supplement: Table S1 — A. baumannii genome sequences used in this study. (DOCX) [file pone.0077631.s001.docx]

**Table S1 -** *A. baumannii* genome sequences used in this study

| **Strain** | **BioProject** | **Status** | **Plasmids** | **Reference** |
| --- | --- | --- | --- | --- |
| AB0057 | PRJNA59083 | Complete | 1 | [[1](#_ENREF_1)] |
| 1656-2 | PRJNA158677 | Complete | 2 |  |
| AB307-0294 | PRJNA59271 | Complete | - | [[1](#_ENREF_1)] |
| ACICU | PRJNA58765 | Complete | 2 | [[2](#_ENREF_2)] |
| ATCC 17978 | PRJNA58731 | Complete | 2 | [[3](#_ENREF_3)] |
| AYE | PRJNA61637 | Complete | 4 | [[4](#_ENREF_4),[5](#_ENREF_5)] |
| MDR-TJ | PRJNA162739 | Complete | 1 | [[6](#_ENREF_6)] |
| MDR-ZJ06 | PRJNA158685 | Complete | 1 | [[7](#_ENREF_7)] |
| SDF | PRJNA61601 | Complete | 3 | [[4](#_ENREF_4),[5](#_ENREF_5)] |
| TCDC-AB0715 | PRJNA158679 | Complete | 2 | [[8](#_ENREF_8)] |
| 3909 | PRJNA52397 | Drafts | 1 | [[9](#_ENREF_9),[10](#_ENREF_10)] |
| 3990 | PRJNA52395 | Drafts | 2 | [[9](#_ENREF_9),[10](#_ENREF_10)] |
| 4190 | PRJNA52399 | Drafts | 2 | [[9](#_ENREF_9),[10](#_ENREF_10)] |
| MS1968 | - | Drafts | - | This study |
| MS1984 | - | Drafts | - | This study |
| 6013113 | PRJNA47501 | Drafts | - |  |
| 6013150 | PRJNA47499 | Drafts | - |  |
| 6014059 | PRJNA47503 | Drafts | - |  |
| A118 | PRJNA60111 | Drafts | - | [[11](#_ENREF_11)] |
| AB056 | PRJNA43427 | Drafts | - | [[12](#_ENREF_12)] |
| AB058 | PRJNA43429 | Drafts | - | [[12](#_ENREF_12)] |
| AB059 | PRJNA43431 | Drafts | - | [[12](#_ENREF_12)] |
| AB210 | PRJNA52391 | Drafts | - | [[13](#_ENREF_13)] |
| AB4857 | PRJNA77019 | Drafts | - | [[14](#_ENREF_14)] |
| AB5075 | PRJNA77021 | Drafts | - | [[14](#_ENREF_14)] |
| AB5256 | PRJNA77023 | Drafts | - | [[14](#_ENREF_14)] |
| AB5711 | PRJNA77025 | Drafts | - | [[14](#_ENREF_14)] |
| AB900 | PRJNA30995 | Drafts | - | [[1](#_ENREF_1)] |
| ABNIH1 | PRJNA63335 | Drafts | - | [[15](#_ENREF_15)] |
| ABNIH2 | PRJNA63337 | Drafts | - | [[15](#_ENREF_15)] |
| ABNIH3 | PRJNA63339 | Drafts | - | [[15](#_ENREF_15)] |
| ABNIH4 | PRJNA63341 | Drafts | - | [[15](#_ENREF_15)] |
| ATCC 19606 | PRJNA40853 | Drafts | - |  |
| D1279779 | PRJNA61919 | Drafts | - | [[16](#_ENREF_16)] |
| Naval-18 | PRJNA53393 | Drafts | - |  |
| Naval-81 | PRJNA53395 | Drafts | - |  |
| OIFC032 | PRJNA53379 | Drafts | - |  |
| UMB001 | PRJNA60843 | Drafts | - | [[17](#_ENREF_17)] |
| UMB002 | PRJNA60845 | Drafts | - | [[17](#_ENREF_17)] |
| UMB003 | PRJNA60847 | Drafts | - | [[17](#_ENREF_17)] |
| W6976 | PRJNA62419 | Drafts | - |  |
| W7282 | PRJNA62421 | Drafts | - |  |
| WM99c | PRJNA61917 | Drafts | - | [[16](#_ENREF_16)] |

1. Adams MD, Goglin K, Molyneaux N, Hujer KM, Lavender H, et al. (2008) Comparative genome sequence analysis of multidrug-resistant Acinetobacter baumannii. J Bacteriol 190: 8053-8064.

2. Iacono M, Villa L, Fortini D, Bordoni R, Imperi F, et al. (2008) Whole-genome pyrosequencing of an epidemic multidrug-resistant Acinetobacter baumannii strain belonging to the European clone II group. Antimicrob Agents Chemother 52: 2616-2625.

3. Smith MG, Gianoulis TA, Pukatzki S, Mekalanos JJ, Ornston LN, et al. (2007) New insights into Acinetobacter baumannii pathogenesis revealed by high-density pyrosequencing and transposon mutagenesis. Genes Dev 21: 601-614.

4. Vallenet D, Nordmann P, Barbe V, Poirel L, Mangenot S, et al. (2008) Comparative analysis of Acinetobacters: three genomes for three lifestyles. PLoS One 3: e1805.

5. Fournier PE, Vallenet D, Barbe V, Audic S, Ogata H, et al. (2006) Comparative genomics of multidrug resistance in Acinetobacter baumannii. PLoS Genet 2: e7.

6. Gao F, Wang Y, Liu YJ, Wu XM, Lv X, et al. (2011) Genome sequence of Acinetobacter baumannii MDR-TJ. J Bacteriol 193: 2365-2366.

7. Zhou H, Zhang T, Yu D, Pi B, Yang Q, et al. (2011) Genomic analysis of the multidrug-resistant Acinetobacter baumannii strain MDR-ZJ06 widely spread in China. Antimicrob Agents Chemother 55: 4506-4512.

8. Chen CC, Lin YC, Sheng WH, Chen YC, Chang SC, et al. (2011) Genome sequence of a dominant, multidrug-resistant Acinetobacter baumannii strain, TCDC-AB0715. J Bacteriol 193: 2361-2362.

9. Di Nocera PP, Rocco F, Giannouli M, Triassi M, Zarrilli R (2011) Genome organization of epidemic Acinetobacter baumannii strains. BMC Microbiol 11: 224.

10. Zarrilli R, Giannouli M, Rocco F, Loman NJ, Haines AS, et al. (2011) Genome sequences of three Acinetobacter baumannii strains assigned to the multilocus sequence typing genotypes ST2, ST25, and ST78. J Bacteriol 193: 2359-2360.

11. Ramirez MS, Adams MD, Bonomo RA, Centron D, Tolmasky ME (2011) Genomic analysis of Acinetobacter baumannii A118 by comparison of optical maps: identification of structures related to its susceptibility phenotype. Antimicrob Agents Chemother 55: 1520-1526.

12. Adams MD, Chan ER, Molyneaux ND, Bonomo RA (2010) Genomewide analysis of divergence of antibiotic resistance determinants in closely related isolates of Acinetobacter baumannii. Antimicrob Agents Chemother 54: 3569-3577.

13. Hornsey M, Loman N, Wareham DW, Ellington MJ, Pallen MJ, et al. (2011) Whole-genome comparison of two Acinetobacter baumannii isolates from a single patient, where resistance developed during tigecycline therapy. J Antimicrob Chemother 66: 1499-1503.

14. Zurawski DV, Thompson MG, McQueary CN, Matalka MN, Sahl JW, et al. (2012) Genome sequences of four divergent multidrug-resistant Acinetobacter baumannii strains isolated from patients with sepsis or osteomyelitis. J Bacteriol 194: 1619-1620.

15. Snitkin ES, Zelazny AM, Montero CI, Stock F, Mijares L, et al. (2011) Genome-wide recombination drives diversification of epidemic strains of Acinetobacter baumannii. Proc Natl Acad Sci U S A 108: 13758-13763.

16. Eijkelkamp BA, Stroeher UH, Hassan KA, Papadimitrious MS, Paulsen IT, et al. (2011) Adherence and motility characteristics of clinical Acinetobacter baumannii isolates. FEMS Microbiol Lett 323: 44-51.

17. Sahl JW, Johnson JK, Harris AD, Phillippy AM, Hsiao WW, et al. (2011) Genomic comparison of multi-drug resistant invasive and colonizing Acinetobacter baumannii isolated from diverse human body sites reveals genomic plasticity. BMC Genomics 12: 291.
