# Supplementary material for: Identification of Novel Vaccine Candidates against Multidrug-Resistant Acinetobacter baumannii
Source: PLoS One. 2013 Oct 8;8(10):e77631. doi: 10.1371/journal.pone.0077631 (PMC3792912; doi:10.1371/journal.pone.0077631)
Supplement: Table S2 — A. baumannii antigens identified through the reverse vaccinology approach. (DOCX) [file pone.0077631.s002.docx]

**Table S2 -** *A. baumannii* antigens identified through the reverse vaccinology approach

| **Locus tag** | **Product** | **Length (aa)** | **SignalP** | **LipoP** | **PSORTb** |
| --- | --- | --- | --- | --- | --- |
| ABAYE0017 | hypothetical protein | 233 | + | + | Outer Membrane |
| ABAYE0041 | hypothetical protein | 135 | - | - | Unkown |
| ABAYE0057 | lipoprotein-34 precursor (NlpB) | 202 | - | + | Unkown |
| ABAYE0070 | hypothetical protein | 228 | - | + | Unkown |
| ABAYE0080 | signal peptide | 609 | + | + | Unkown Multiple Sites |
| ABAYE0113 | hypothetical protein | 195 | - | + | Unkown |
| ABAYE0120 | hypothetical protein | 153 | - | + | Unkown |
| ABAYE0137 | hypothetical protein | 260 | + | + | Unkown |
| ABAYE0141 | hypothetical protein | 146 | + | + | Unkown |
| ABAYE0145 | ferric siderophore receptor protein | 737 | + | + | Outer Membrane |
| ABAYE0156 | hypothetical protein | 231 | + | + | Unkown |
| ABAYE0170 | hypothetical protein | 256 | + | + | Outer Membrane |
| ABAYE0191 | hypothetical protein | 301 | + | + | Outer Membrane |
| ABAYE0220 | signal peptide | 248 | + | + | Unkown |
| ABAYE0228 | hypothetical protein | 180 | - | + | Unkown |
| ABAYE0266 | signal peptide | 198 | - | + | Unkown |
| ABAYE0267 | signal peptide | 233 | - | + | Unkown |
| ABAYE0304 | fimbrial protein precursor (pilin) | 158 | - | - | Extracellular |
| ABAYE0318 | competence factor involved in DNA binding and | 1289 | - | + | Extracellular |
| ABAYE0337 | signal peptide | 127 | - | + | Unkown |
| ABAYE0346 | hypothetical protein | 181 | - | + | Unkown |
| ABAYE0358 | signal peptide | 104 | - | + | Unkown |
| ABAYE0359 | alkaline protease | 462 | + | + | Extracellular |
| ABAYE0360 | signal peptide | 144 | + | + | Extracellular |
| ABAYE0361 | signal peptide | 140 | + | + | Unkown |
| ABAYE0374 | signal peptide | 219 | - | + | Unkown |
| ABAYE0439 | hypothetical protein | 362 | - | + | Unkown |
| ABAYE0448 | hypothetical protein | 808 | - | - | Extracellular |
| ABAYE0492 | signal peptide | 150 | + | + | Unkown |
| ABAYE0500 | lipoprotein precursor | 160 | - | + | Outer Membrane |
| ABAYE0506 | hypothetical protein | 402 | - | + | Unkown |
| ABAYE0591 | TonB-dependent receptor protein | 692 | + | + | Outer Membrane |
| ABAYE0594 | signal peptide | 128 | + | + | Unkown |
| ABAYE0606 | TonB-dependent Outer membrane receptor for | 639 | + | + | Outer Membrane |
| ABAYE0640 | Outer membrane protein precursor (OmpA-like) | 354 | + | + | Outer Membrane |
| ABAYE0653 | tonB-dependent receptor protein | 735 | + | + | Outer Membrane |
| ABAYE0656 | hypothetical protein | 275 | + | + | Unkown |
| ABAYE0693 | hypothetical protein | 250 | - | + | Unkown |
| ABAYE0695 | hypothetical protein | 173 | + | + | Unkown |
| ABAYE0790 | hypothetical protein | 300 | - | - | Unkown |
| ABAYE0792 | hypothetical protein | 8201 | - | - | Unkown Multiple Sites |
| ABAYE0795 | metalloprotease | 246 | - | + | Unkown |
| ABAYE0811 | alkaline phosphatase D precursor | 588 | + | + | Extracellular |
| ABAYE0814 | hypothetical protein | 141 | + | + | Unkown |
| ABAYE0821 | hypothetical protein | 3370 | - | - | Unkown Multiple Sites |
| ABAYE0892 | signal peptide | 627 | + | + | Unkown |
| ABAYE0893 | outer membrane protein | 817 | + | + | Outer Membrane |
| ABAYE0908 | hydrolase | 445 | + | + | Unkown |
| ABAYE0917 | signal peptide | 190 | - | + | Unkown |
| ABAYE0924 | porin protein associated with imipenem | 247 | + | + | Unkown |
| ABAYE0964 | hypothetical protein | 403 | + | + | Unkown |
| ABAYE0990 | protease | 921 | + | + | Outer Membrane |
| ABAYE1024 | hypothetical protein | 234 | - | + | Unkown |
| ABAYE1037 | hypothetical protein | 729 | - | - | Extracellular |
| ABAYE1045 | hypothetical protein | 141 | + | + | Unkown |
| ABAYE1048 | lipoprotein precursor | 277 | - | + | Outer Membrane |
| ABAYE1070 | signal peptide | 391 | + | + | Outer Membrane |
| ABAYE1093 | ferric acinetobactin receptor (bauA) | 768 | + | - | Outer Membrane |
| ABAYE1108 | signal peptide | 157 | + | + | Unkown |
| ABAYE1109 | signal peptide | 632 | + | + | Unkown |
| ABAYE1121 | TonB-dependent receptor | 925 | - | - | Outer Membrane |
| ABAYE1122 | aminopeptidase N(PEPN) | 923 | + | + | Unkown Multiple Sites |
| ABAYE1129 | hydrolase | 486 | + | + | Unkown |
| ABAYE1151 | hypothetical protein | 373 | + | + | Unkown |
| ABAYE1197 | hypothetical protein | 184 | - | + | Unkown |
| ABAYE1221 | hypothetical protein | 127 | - | + | Unkown |
| ABAYE1319 | protein CsuA/B; secreted protein related to type | 181 | + | + | Unkown |
| ABAYE1320 | protein CsuA | 193 | - | - | Unkown |
| ABAYE1321 | protein CsuB; secreted protein related to type I | 173 | - | + | Extracellular |
| ABAYE1394 | biofilm synthesis protein | 813 | - | + | Unkown Multiple Sites |
| ABAYE1395 | biofilm synthesis protein | 610 | + | + | Unkown |
| ABAYE1401 | hypothetical protein | 176 | + | + | Unkown |
| ABAYE1429 | hypothetical protein | 907 | - | + | Outer Membrane |
| ABAYE1470 | biofilm synthesis protein | 177 | + | + | Extracellular |
| ABAYE1473 | hypothetical protein | 340 | - | + | Extracellular |
| ABAYE1486 | siderophore receptor | 757 | + | + | Outer Membrane |
| ABAYE1494 | outer membrane porin, receptor for | 719 | + | + | Outer Membrane |
| ABAYE1520 | phospholipase C precursor (PLC) | 745 | + | + | Extracellular |
| ABAYE1536 | hypothetical protein | 168 | + | + | Unkown |
| ABAYE1583 | outer membrane protein | 842 | + | + | Outer Membrane |
| ABAYE1602 | extracellular serine proteinase | 397 | + | + | Extracellular |
| ABAYE1634 | hypothetical protein | 224 | + | + | Unkown |
| ABAYE1644 | ferrisiderophore receptor protein, TonB | 700 | - | + | Outer Membrane |
| ABAYE1646 | phospholipase A1 precursor (PldA) | 384 | + | + | Outer Membrane |
| ABAYE1652 | hypothetical protein | 507 | - | + | Extracellular |
| ABAYE1666 | signal peptide | 168 | + | + | Unkown |
| ABAYE1686 | signal peptide | 163 | - | + | Unkown |
| ABAYE1753 | hypothetical protein | 242 | - | + | Unkown |
| ABAYE1763 | hypothetical protein | 104 | - | + | Unkown |
| ABAYE1802 | acid phosphatase | 324 | + | + | Extracellular |
| ABAYE1803 | signal peptide | 215 | + | + | Unkown |
| ABAYE1856 | fimbrial protein precursor (pilin) | 178 | + | + | Extracellular |
| ABAYE1859 | fimbria adhesin protein | 337 | - | + | Extracellular |
| ABAYE1860 | hypothetical protein | 326 | - | + | Outer Membrane |
| ABAYE1922 | ferric siderophore receptor protein | 735 | + | + | Outer Membrane |
| ABAYE1979 | porin precursor | 369 | + | + | Outer Membrane |
| ABAYE1984 | ferric siderophore receptor protein | 722 | + | + | Outer Membrane |
| ABAYE1991 | hypothetical protein | 523 | - | + | Outer Membrane |
| ABAYE2001 | ferric siderophore receptor protein | 773 | - | + | Outer Membrane |
| ABAYE2043 | metallopeptidase | 678 | + | + | Unkown Multiple Sites |
| ABAYE2046 | outer membrane protein; TonB-dependent receptor | 905 | + | + | Outer Membrane |
| ABAYE2112 | signal peptide | 252 | - | + | Unkown |
| ABAYE2114 | hypothetical protein | 197 | + | + | Unkown |
| ABAYE2123 | antibiotic resistance (phosphinothricin | 182 | - | - | Unkown |
| ABAYE2132 | fimbrial protein precursor (pilin) | 210 | + | + | Extracellular |
| ABAYE2138 | fimbria adhesin protein | 342 | - | + | Extracellular |
| ABAYE2174 | hypothetical protein | 112 | + | + | Unkown |
| ABAYE2192 | hypothetical protein | 314 | - | + | Unkown |
| ABAYE2274 | hypothetical protein | 106 | - | - | Unkown |
| ABAYE2330 | hypothetical protein | 390 | - | + | Outer Membrane |
| ABAYE2357 | hypothetical protein | 209 | - | + | Unkown |
| ABAYE2389 | hypothetical protein | 145 | + | + | Unkown |
| ABAYE2413 | hypothetical protein | 168 | - | - | Extracellular |
| ABAYE2436 | hypothetical protein | 303 | - | + | Extracellular |
| ABAYE2447 | hypothetical protein | 236 | - | - | Unkown |
| ABAYE2463 | hypothetical protein | 544 | - | + | Unkown |
| ABAYE2464 | hypothetical protein | 146 | - | + | Unkown |
| ABAYE2498 | hypothetical protein | 197 | - | + | Unkown |
| ABAYE2523 | hypothetical protein | 210 | - | - | Unkown |
| ABAYE2524 | hypothetical protein | 198 | + | + | Unkown |
| ABAYE2569 | hypothetical protein | 115 | + | + | Unkown |
| ABAYE2585 | M24/M37 family peptidase | 273 | + | + | Unkown |
| ABAYE2590 | hypothetical protein | 260 | + | + | Unkown |
| ABAYE2648 | TonB-dependent siderophore receptor precursor | 706 | + | + | Outer Membrane |
| ABAYE2653 | Rhs family protein | 1600 | + | + | Unkown Multiple Sites |
| ABAYE2655 | hypothetical protein | 110 | - | + | Unkown |
| ABAYE2700 | hypothetical protein | 230 | - | + | Unkown |
| ABAYE2750 | hypothetical protein | 156 | + | + | Unkown |
| ABAYE2757 | signal peptide | 106 | + | + | Unkown |
| ABAYE2758 | signal peptide | 117 | + | + | Unkown |
| ABAYE2781 | hypothetical protein | 108 | - | + | Unkown |
| ABAYE2782 | lipoprotein | 160 | + | + | Outer Membrane |
| ABAYE2793 | hypothetical protein | 482 | + | + | Outer Membrane |
| ABAYE2812 | outer membrane receptor FepA | 755 | + | + | Outer Membrane |
| ABAYE2835 | hypothetical protein | 399 | + | + | Outer Membrane |
| ABAYE2921 | outer membrane lipoprotein | 133 | - | + | Outer Membrane |
| ABAYE2966 | hypothetical protein | 395 | + | + | Outer Membrane |
| ABAYE2977 | hypothetical protein | 570 | + | + | Unkown |
| ABAYE2985 | hypothetical protein | 187 | + | + | Unkown |
| ABAYE3021 | large exoproteins involved in heme utilization | 817 | + | + | Unkown Multiple Sites |
| ABAYE3024 | hypothetical protein | 459 | + | + | Unkown |
| ABAYE3068 | Outer Membrane protein exposed to the surface | 975 | - | + | Extracellular |
| ABAYE3128 | pilus subunit (FilA) | 283 | + | + | Extracellular |
| ABAYE3138 | lipoprotein precursor (VacJ) transmembrane | 300 | + | + | Outer Membrane |
| ABAYE3230 | hypothetical protein | 315 | - | + | Unkown |
| ABAYE3231 | hypothetical protein | 281 | - | + | Unkown |
| ABAYE3245 | minor lipoprotein | 170 | + | + | Unkown |
| ABAYE3286 | hypothetical protein | 239 | - | + | Unkown |
| ABAYE3290 | ferric siderophore receptor protein | 744 | + | + | Outer Membrane |
| ABAYE3295 | hypothetical protein | 196 | + | + | Unkown |
| ABAYE3334 | signal peptide | 253 | + | + | Unkown |
| ABAYE3358 | hypothetical protein | 197 | - | + | Unkown |
| ABAYE3452 | hypothetical protein | 442 | - | - | Unkown |
| ABAYE3468 | hypothetical protein | 334 | - | + | Outer Membrane |
| ABAYE3478 | hypothetical protein | 380 | - | + | Outer Membrane |
| ABAYE3672 | hypothetical protein | 271 | + | + | Outer Membrane |
| ABAYE3703 | outer membrane copper receptor (OprC) | 705 | + | + | Outer Membrane |
| ABAYE3706 | hypothetical protein | 385 | - | + | Unkown |
| ABAYE3714 | hypothetical protein | 209 | - | + | Unkown |
| ABAYE3745 | hypothetical protein | 138 | + | + | Unkown |
| ABAYE3777 | ferric siderophore receptor protein | 697 | - | + | Outer Membrane |
| ABAYE3819 | FKBP-type peptidyl-prolyl cis-trans isomerase | 241 | - | + | Outer Membrane |
| ABAYE3820 | FKBP-type 22KD peptidyl-prolyl cis-trans | 236 | + | + | Outer Membrane |
| ABAYE3825 | phospholipase C precursor (PLC) | 738 | - | - | Extracellular |
| ABAYE3837 | hypothetical protein | 163 | + | + | Unkown |
| ABAYE3866 | peptidase | 306 | - | + | Outer Membrane |
| ABAYE3867 | hypothetical protein | 337 | + | - | Unkown |
| ABAYE3882 | signal peptide | 126 | - | + | Unkown |
| ACICU_00110 | TPR repeat-containing SEL1 subfamily protein | 231 | - | + | Extracellular |
| ACICU_00530 | hypothetical protein | 182 | - | + | Unkown |
| ACICU_00744 | hypothetical protein | 502 | + | + | Unkown |
| ACICU_00875 | outer membrane receptor | 1069 | + | + | Outer Membrane |
| ACICU_00876 | hypothetical protein | 264 | + | + | Extracellular |
| ACICU_00877 | hypothetical protein | 497 | + | + | Outer Membrane |
| ACICU_00993 | autotransporter adhesin | 1863 | + | + | Outer Membrane |
| ACICU_01059 | hypothetical protein | 314 | + | + | Unkown |
| ACICU_01069 | outer membrane receptor | 191 | + | + | Unkown |
| ACICU_01071 | outer membrane receptor | 443 | - | - | Outer Membrane |
| ACICU_01074 | hypothetical protein | 163 | - | + | Unkown |
| ACICU_01118 | Rhs family protein | 1566 | - | - | Outer Membrane |
| ACICU_01119 | TPR repeat-containing SEL1 subfamily protein | 286 | - | + | Extracellular |
| ACICU_01120 | TPR repeat-containing SEL1 subfamily protein | 242 | - | + | Extracellular |
| ACICU_01121 | TPR repeat-containing SEL1 subfamily protein | 160 | - | + | Unkown |
| ACICU_01854 | hypothetical protein | 185 | - | + | Unkown |
| ACICU_01891 | RTX toxin | 1451 | - | - | Extracellular_Multiple Sites |
| ACICU_01893 | putative DcaP-like protein | 423 | - | + | Outer Membrane |
| ACICU_01911 | large exoprotein | 2142 | - | - | Outer Membrane |
| ACICU_01912 | hemolysin activation/secretion protein | 582 | - | + | Outer Membrane |
| ACICU_01931 | esterase/lipase | 337 | + | + | Unkown Multiple Sites |
| ACICU_02200 | hypothetical protein | 352 | + | + | Unkown Multiple Sites |
| ACICU_02527 | outer membrane protein W | 357 | + | + | Unkown |
| ACICU_02736 | Ig-like domain-containing surface protein | 319 | - | - | Unkown |
| ACICU_02845 | putative outer membrane protein A | 557 | + | + | Unkown |
| ACICU_02938 | hemolysin-type calcium-binding domain-containing | 2140 | - | - | Unkown Multiple Sites |
| ACICU_02966 | hypothetical protein | 278 | + | + | Unkown |
| ACICU_03124 | outer membrane cobalamin receptor protein | 628 | + | + | Outer Membrane |
| ABK1_0173 | hypothetical protein | 148 | + | + | Unkown |
| ABK1_1372 | hypothetical protein | 191 | - | + | Unkown |
| ABK1_1548 | hypothetical protein | 174 | - | + | Unkown |
| ABK1_1555 | hypothetical protein | 273 | - | + | Unkown |
| A1S_0474 | putative ferric siderophore receptor protein | 737 | - | - | Outer Membrane |
| A1S_0634 | hypothetical protein | 626 | + | + | Unkown |
| A1S_0742 | iron-regulated protein | 897 | - | + | Extracellular_Multiple Sites |
| A1S_0785 | hypothetical protein | 417 | - | - | Unkown |
| A1S_0911 | hypothetical protein | 201 | - | + | Unkown |
| A1S_1073 | hemolysin-type calcium-binding region | 3217 | - | - | Extracellular_Multiple Sites |
| A1S_2385 | putative ferric acinetobactin receptor | 735 | - | + | Outer Membrane |
| A1S_2413 | putative hemolysin-type calcium-binding region | 443 | - | - | Extracellular |
| A1S_2414 | putative calcium binding hemolysin protein | 595 | - | - | Extracellular |
| A1S_2563 | putative siderophore-interacting protein | 202 | - | - | Unkown |
| A1S_2566 | putative ferric siderophore receptor protein | 553 | - | - | Outer Membrane |
| A1S_2601 | putative outer membrane protein A | 797 | - | - | Unkown |
| A1S_3177 | fimbrial protein | 155 | - | - | Extracellular |
| A1S_3273 | putative peptide signal | 128 | + | + | Unkown |
| AB57_2033 | hypothetical protein | 431 | + | + | Unkown |
| ABBFA_000233 | hypothetical protein | 150 | + | + | Extracellular |
| ABBFA_002519 | hypothetical protein | 174 | - | + | Unkown |
| ABBFA_002521 | hypothetical protein | 225 | + | + | Unkown |
| ABBFA_002533 | hypothetical protein | 228 | - | + | Unkown |
| ABTJ_00117 | metalloendopeptidase-like membrane protein | 532 | - | - | Unkown |
| ABTJ_00224 | Sel1 repeat protein | 188 | + | + | Extracellular |
| ABTJ_00226 | hypothetical protein | 294 | - | + | Unkown |
| ABTJ_01549 | hypothetical protein | 119 | + | + | Unkown |
| ABZJ_00944 | hypothetical protein | 274 | - | + | Unkown |
| ABSDF0703 | hypothetical protein | 306 | - | - | Unkown |
| ABSDF0785 | cell-surface adhesin | 2322 | - | - | Outer Membrane |
| ABSDF1384 | hemolysin activator protein | 579 | - | + | Outer Membrane |
| ABSDF1812 | hypothetical protein | 163 | - | + | Unkown |
| ABSDF2314 | cell-surface adhesin | 2403 | - | - | Extracellular |
| ABSDF2466 | hypothetical protein | 232 | + | + | Unkown |
| ABSDF2467 | hypothetical protein | 129 | + | + | Unkown |
| ABSDF2492 | hypothetical protein | 153 | - | - | Unkown |
| ABSDF3544 | hemagglutinin/hemolysin-related protein | 4087 | - | - | Outer Membrane |
| ABTW07_3037 | hypothetical protein | 110 | - | + | Unkown |
