# Supplementary material for: Identification of Novel Vaccine Candidates against Multidrug-Resistant Acinetobacter baumannii
Source: PLoS One. 2013 Oct 8;8(10):e77631. doi: 10.1371/journal.pone.0077631 (PMC3792912; doi:10.1371/journal.pone.0077631)
Supplement: Table S3 — A. baumannii OMV and secretome. (DOCX) [file pone.0077631.s003.docx]

**Table S3 -** *A. baumannii* OMV and secretome

| **Locus tag** | **Product** | **Length (aa)** | **SignalP** | **LipoP** | **PSORTb** | **OMV** | **S^a^** |
| --- | --- | --- | --- | --- | --- | --- | --- |
| ABAYE0235 | Signal peptide | 164 | + | + | Extracellular | + |  |
| ABAYE0304 | Fimbrial protein precursor (pilin) | 158 | - | - | Extracellular | + | + |
| ABAYE0360 | Signal peptide | 144 | + | + | Extracellular | + | + |
| ABAYE0448 | Hypothetical protein | 808 | - | - | Extracellular | + | + |
| ABAYE1037 | Hypothetical protein | 729 | - | - | Extracellular | + | + |
| ABAYE1470 | Biofilm synthesis protein | 177 | + | + | Extracellular |  | + |
| ABAYE1588 | Hypothetical protein | 295 | - | + | Extracellular | + |  |
| ABAYE1856 | Fimbrial protein precursor (pilin) | 178 | + | + | Extracellular | + | + |
| ABAYE1859 | Fimbria adhesin protein | 337 | - | + | Extracellular | + | + |
| ABAYE2132 | Fimbrial protein precursor (pilin) | 210 | + | + | Extracellular |  | + |
| ABAYE3267 | Nucleoside diphosphate kinase | 144 | - | - | Extracellular | + |  |
| ACICU_01891 | RTX toxin | 1451 | - | - | Extracellular (Multiple sites) |  | + |
| ABAYE0017 | Hypothetical protein | 233 | + | + | Outer Membrane | + |  |
| ABAYE0130 | Hypothetical protein | 242 | + | + | Outer Membrane | + |  |
| ABAYE0145 | Ferric siderophore receptor protein | 737 | + | + | Outer Membrane | + |  |
| ABAYE0170 | Hypothetical protein | 256 | + | + | Outer Membrane | + | + |
| ABAYE0191 | Hypothetical protein | 301 | + | + | Outer Membrane | + |  |
| ABAYE0500 | Lipoprotein precursor | 160 | - | + | Outer Membrane | + |  |
| ABAYE0606 | TonB-dependent Outer membrane receptor for | 639 | + | + | Outer Membrane | + |  |
| ABAYE0632 | Glucose-sensitive porin (OprB-like ) | 418 | + | + | Outer Membrane | + |  |
| ABAYE0640 | Outer membrane protein precursor (OmpA-like) | 354 | + | + | Outer Membrane | + | + |
| ABAYE0730 | Hypothetical protein | 436 | + | + | Outer Membrane | + |  |
| ABAYE0746 | Outer membrane protein (AdeC-like) | 486 | + | + | Outer Membrane | + |  |
| ABAYE0901 | Peptidoglycan-associated lipoprotein precursor | 192 | - | + | Outer Membrane | + |  |
| ABAYE0990 | Protease | 921 | + | + | Outer Membrane | + |  |
| ABAYE1048 | Lipoprotein precursor | 277 | - | + | Outer Membrane | + |  |
| ABAYE1093 | Ferric acinetobactin receptor (bauA) | 768 | + | - | Outer Membrane | + |  |
| ABAYE1486 | Siderophore receptor | 757 | + | + | Outer Membrane | + |  |
| ABAYE1494 | Outer membrane porin, receptor for | 719 | + | + | Outer Membrane | + |  |
| ABAYE1583 | Outer membrane protein | 842 | + | + | Outer Membrane | + |  |
| ABAYE1644 | Ferrisiderophore receptor protein, TonB | 700 | - | + | Outer Membrane | + |  |
| ABAYE1646 | Phospholipase A1 precursor (PldA) | 384 | + | + | Outer Membrane | + |  |
| ABAYE1860 | Hypothetical protein | 326 | - | + | Outer Membrane |  | + |
| ABAYE1984 | Ferric siderophore receptor protein | 722 | + | + | Outer Membrane | + |  |
| ABAYE2001 | Ferric siderophore receptor protein | 773 | - | + | Outer Membrane | + |  |
| ABAYE2663 | Bifunctional protein [Includes: lytic murein | 1072 | + | + | Outer Membrane | + |  |
| ABAYE2793 | Hypothetical protein | 482 | + | + | Outer Membrane | + |  |
| ABAYE2812 | Outer membrane receptor FepA | 755 | + | + | Outer Membrane | + | + |
| ABAYE2921 | Outer membrane lipoprotein | 133 | - | + | Outer Membrane | + |  |
| ABAYE2931 | Outer membrane protein | 218 | - | + | Outer Membrane | + | + |
| ABAYE2974 | Competence protein (ComL) | 386 | - | + | Outer Membrane | + |  |
| ABAYE3123 | Pilus assembly protein (FilF) | 642 | - | + | Outer Membrane | + | + |
| ABAYE3126 | Pilus assembly protein (FilC) | 406 | - | + | Outer Membrane | + |  |
| ABAYE3265 | Type 4 fimbrial biogenesis protein | 267 | + | + | Outer Membrane | + |  |
| ABAYE3290 | Ferric siderophore receptor protein | 744 | + | + | Outer Membrane | + | + |
| ABAYE3468 | Hypothetical protein | 334 | - | + | Outer Membrane | + | + |
| ABAYE3478 | Hypothetical protein | 380 | - | + | Outer Membrane | + |  |
| ABAYE3482 | Outer membrane protein W | 207 | + | + | Outer Membrane | + |  |
| ABAYE3674 | Outer membrane protein | 439 | + | + | Outer Membrane | + |  |
| ABAYE3703 | Outer membrane copper receptor (OprC) | 705 | + | + | Outer Membrane | + |  |
| ACICU_01911 | Large exoprotein | 2142 | - | - | Outer Membrane |  | + |
| ACICU_03521 | Outer membrane receptor | 719 | + | + | Outer Membrane | + |  |
| A1S_1164 | Putative phage tail tape meausure protein | 623 | - | - | Unknown | + |  |
| A1S_1517 | Beta-lactamase OXA-95 | 192 | - | - | Unknown | + |  |
| AB57_0551 | Beta-lactamase OXA-23 | 274 | - | + | Unknown | + |  |
| ABAYE0009 | RND type efflux pump involved in aminoglycoside | 336 | + | + | Unknown | + |  |
| ABAYE0057 | Lipoprotein-34 precursor (NlpB) | 202 | - | + | Unknown | + |  |
| ABAYE0120 | Hypothetical protein | 153 | - | + | Unknown | + |  |
| ABAYE0266 | Signal peptide | 198 | - | + | Unknown | + |  |
| ABAYE0267 | Signal peptide | 233 | - | + | Unknown | + |  |
| ABAYE0334 | Hypothetical protein | 126 | - | + | Unknown | + | + |
| ABAYE0388 | Toluene tolerance protein (Ttg2D) | 220 | + | + | Unknown | + |  |
| ABAYE0404 | Nucleotide triphosphate hydrolase | 516 | - | - | Unknown | + |  |
| ABAYE0465 | Malate dehydrogenase | 329 | - | - | Unknown | + | + |
| ABAYE0506 | Hypothetical protein | 402 | - | + | Unknown | + |  |
| ABAYE0603 | Signal peptide | 189 | - | + | Unknown | + |  |
| ABAYE0795 | Metalloprotease | 246 | - | + | Unknown | + |  |
| ABAYE0814 | Hypothetical protein | 141 | + | + | Unknown | + |  |
| ABAYE0825 | Metallo-beta-lactamase | 321 | + | + | Unknown | + | + |
| ABAYE0866 | Electron transfer flavoprotein alpha-subunit | 311 | - | - | Unknown | + |  |
| ABAYE0920 | Hypothetical protein | 139 | + | + | Unknown | + |  |
| ABAYE0924 | Porin protein associated with imipenem | 247 | + | + | Unknown | + |  |
| ABAYE0943 | Hypothetical protein | 67 | - | + | Unknown | + |  |
| ABAYE1045 | Hypothetical protein | 141 | + | + | Unknown | + |  |
| ABAYE1105 | Hypothetical protein | 419 | + | + | Unknown | + | + |
| ABAYE1129 | Hydrolase | 486 | + | + | Unknown | + | + |
| ABAYE1197 | Hypothetical protein | 184 | - | + | Unknown | + |  |
| ABAYE1205 | Hypothetical protein | 315 | + | + | Unknown | + |  |
| ABAYE1221 | Hypothetical protein | 127 | - | + | Unknown | + | + |
| ABAYE1269 | Hypothetical protein | 120 | - | + | Unknown | + |  |
| ABAYE1298 | Hypothetical protein | 78 | - | - | Unknown | + | + |
| ABAYE1303 | Signal peptide | 121 | + | + | Unknown | + |  |
| ABAYE1319 | Protein CsuA/B; secreted protein related to type | 181 | + | + | Unknown |  | + |
| ABAYE1584 | Outer membrane protein (OmpH) | 168 | + | + | Unknown | + |  |
| ABAYE1605 | Quinoprotein glucose dehydrogenase-B precursor | 481 | + | + | Unknown | + |  |
| ABAYE1611 | Signal peptide | 169 | - | + | Unknown | + |  |
| ABAYE1633 | Hypothetical protein | 199 | - | - | Unknown | + |  |
| ABAYE1686 | Signal peptide | 163 | - | + | Unknown | + |  |
| ABAYE1973 | Hypothetical protein | 125 | - | + | Unknown | + |  |
| ABAYE1974 | Signal peptide | 78 | - | + | Unknown | + |  |
| ABAYE1977 | Hypothetical protein | 90 | + | + | Unknown | + |  |
| ABAYE2114 | Hypothetical protein | 197 | + | + | Unknown | + |  |
| ABAYE2178 | Hypothetical protein | 130 | - | - | Unknown | + |  |
| ABAYE2192 | Hypothetical protein | 314 | - | + | Unknown | + |  |
| ABAYE2267 | Hypothetical protein | 412 | - | - | Unknown |  | + |
| ABAYE2389 | Hypothetical protein | 145 | + | + | Unknown | + | + |
| ABAYE2498 | Hypothetical protein | 197 | - | + | Unknown | + |  |
| ABAYE2501 | Hypothetical protein | 258 | - | + | Unknown |  | + |
| ABAYE2552 | Copper chaperone | 67 | - | - | Unknown | + |  |
| ABAYE2590 | Hypothetical protein | 260 | + | + | Unknown | + |  |
| ABAYE2700 | Hypothetical protein | 230 | - | + | Unknown | + |  |
| ABAYE2763 | Hypothetical protein | 370 | + | + | Unknown | + |  |
| ABAYE2978 | Outer-membrane lipoprotein precursor | 193 | - | + | Unknown | + |  |
| ABAYE2990 | LysM domain/BON superfamily protein | 158 | - | - | Unknown |  | + |
| ABAYE3028 | MTA/SAH nucleosidase [Includes: | 291 | + | + | Unknown | + |  |
| ABAYE3030 | Hypothetical protein | 172 | - | - | Unknown | + |  |
| ABAYE3245 | Minor lipoprotein | 170 | + | + | Unknown | + |  |
| ABAYE3358 | Hypothetical protein | 197 | - | + | Unknown | + |  |
| ABAYE3531 | Threonine synthase | 380 | - | - | Unknown | + |  |
| ABAYE3745 | Hypothetical protein | 138 | + | + | Unknown | + |  |
| ABTJ_00118 | Hypothetical protein | 233 | - | + | Unknown | + |  |
| ACICU_01150 | Hypothetical protein | 130 | + | + | Unknown |  |  |
| ACICU_03056 | Bacteriolytic lipoprotein entericidin B | 47 | - | + | Unknown | + |  |
| ABAYE0792 | Hypothetical protein | 8201 | - | - | Unknown (Multiple sites) | + | + |
| ABAYE0821 | Hypothetical protein | 3370 | - | - | Unknown (Multiple sites) | + | + |
| ABAYE1438 | Signal peptide | 265 | + | + | Unknown (Multiple sites) | + |  |
| ABAYE2043 | Metallopeptidase | 678 | + | + | Unknown (Multiple sites) | + |  |
| ABAYE3260 | Hypothetical protein | 382 | - | + | Unknown (Multiple sites) | + | + |
| ABAYE3490 | 50S ribosomal protein L7/L12 | 124 | + | - | Unknown (Multiple sites) | + | + |
| ABAYE3623 | Beta-lactamase VEB-1 | 300 | + | + | Unknown (Multiple sites) | + | + |
| ABAYE3713 | Glutathione peroxidase | 182 | - | - | Unknown (Multiple sites) | + |  |
| ACICU_01931 | Esterase/lipase | 337 | + | + | Unknown (Multiple sites) | + |  |

^a^Secretome
