# Supplementary material for: Identification of Novel Vaccine Candidates against Multidrug-Resistant Acinetobacter baumannii
Source: PLoS One. 2013 Oct 8;8(10):e77631. doi: 10.1371/journal.pone.0077631 (PMC3792912; doi:10.1371/journal.pone.0077631)
Supplement: Table S4 — A. baumannii potentially insoluble proteins. (DOCX) [file pone.0077631.s004.docx]

**Table S4 -** *A. baumannii* potentially insoluble proteins

| **Locus tag** | **SN** | **OMV** | **Product** | **Length (aa)** | **PSORTb** | **SignalP** | **LipoP** | **Pfam** |
| --- | --- | --- | --- | --- | --- | --- | --- | --- |
| ABAYE0017 | - | + | Hypothetical protein | 233 | Outer Membrane | + | + |  |
| ABAYE0145 | - | + | Ferric siderophore receptor | 737 | Outer Membrane | + | + | TonB dependent receptor (PF00593) |
| ABAYE0170 | + | + | Hypothetical protein | 256 | Outer Membrane | + | + | *S. aureus* Ser-Asp rich fibrinogen-binding protein |
| ABAYE0191 | - | + | Hypothetical protein | 301 | Outer Membrane | + | + | *S. aureus* Ser-Asp rich fibrinogen-binding protein |
| ABAYE0606 | - | + | Outer membrane receptor Btub | 639 | Outer Membrane | + | + | TonB-dependent Receptor Plug Domain (PF07715) |
| ABAYE0640 | + | + | Outer membrane protein precursor (OmpA-like) | 354 | Outer Membrane | + | + | Outer membrane protein beta-barrel domain (PF13505) |
| ABAYE0924 | - | + | Protein associated with imipenem resistance | 247 | Unknown | + | + | OmpW-like |
| ABAYE1093 | - | + | Ferric acinetobactin receptor BauA | 768 | Outer Membrane | + | - | TonB dependent receptor (PF00593) |
| ABAYE1486 | - | + | Siderophore receptor | 757 | Outer Membrane | + | + | TonB dependent receptor (PF00593) |
| ABAYE1494 | - | + | Outer membrane porin FhuE | 719 | Outer Membrane | + | + | TonB dependent receptor (PF00593) |
| ABAYE1583 | - | + | Outer membrane protein | 842 | Outer Membrane | + | + | Surface antigen (PF01103) |
| ABAYE1644 | - | + | Ferrisiderophore receptor | 700 | Outer Membrane | - | + | TonB-dependent Receptor Plug Domain (PF07715) |
| ABAYE1646 | - | + | Phospholipase A1 precursor | 384 | Outer Membrane | + | + | Phospholipase A1 (PF02253) |
| ABAYE1984 | - | + | Ferric siderophore receptor | 722 | Outer Membrane | + | + | TonB-dependent Receptor Plug Domain (PF07715) |
| ABAYE2001 | - | + | Ferric siderophore receptor | 773 | Outer Membrane | - | + | TonB dependent receptor (PF00593) |
| ABAYE2114 | - | + | Hypothetical protein | 197 | Unknown | + | + | YceI-like domain (PF04264) |
| ABAYE2793 | - | + | Hypothetical protein | 482 | Outer Membrane | + | + | Capsule assembly (PF14052) |
| ABAYE2812 | + | + | Outer membrane receptor FepA | 755 | Outer Membrane | + | + | TonB-dependent Receptor Plug Domain (PF07715) |
| ABAYE3290 | + | + | Ferric siderophore receptor | 744 | Outer Membrane | + | + | TonB dependent receptor (PF00593) |
| ABAYE3703 | - | + | Outer membrane copper receptor OprC | 705 | Outer Membrane | + | + | TonB-dependent Receptor Plug Domain (PF07715) |
