# Supplementary material for: Identification of Novel Vaccine Candidates against Multidrug-Resistant Acinetobacter baumannii
Source: PLoS One. 2013 Oct 8;8(10):e77631. doi: 10.1371/journal.pone.0077631 (PMC3792912; doi:10.1371/journal.pone.0077631)
Supplement: Table S5 — A. baumannii periplasmic proteins found in OMV and secretome. (DOCX) [file pone.0077631.s005.docx]

**Table S5 -** *A. baumannii* periplasmic proteins found in OMV and secretome

| **Locus tag** | **Product** | **Length (aa)** | **Signal Peptide** | **PSORTb** | **OMV** | **S^a^** |
| --- | --- | --- | --- | --- | --- | --- |
| ABAYE1110 | Beta-lactamase (ampC) | 397 | + | Periplasmic | + | + |
| ABAYE2087 | Peptidyl-prolyl cis-trans isomerase (SurA) | 442 | + | Periplasmic | + |  |
| ABAYE2829 | Aldose 1-epimerase | 382 | + | Periplasmic | + |  |
| ABAYE0902 | Translocation protein (TolB) | 427 | + | Periplasmic | + |  |
| ABAYE0340 | Superoxide dismutase precursor (Cu-Zn) (sodC) | 206 | + | Periplasmic | + |  |
| ABAYE0936 | Serine protease | 477 | + | Periplasmic | + |  |
| ABAYE0657 | Thiol:disulphide interchange protein (DsbC-like) | 237 | + | Periplasmic | + |  |
| ABAYE3833 | Thiol:disulfide interchange protein (dsbA) | 206 | + | Periplasmic | + |  |
| ABAYE2668 | Hypothetical protein | 236 | + | Periplasmic | + |  |
| ABAYE2161 | Glutamate/aspartate ABC transporter substrate-binding protein (gltI) | 298 | + | Periplasmic | + |  |
| ABAYE0598 | Acid shock protein precursor | 127 | + | Periplasmic | + |  |
| ABAYE1477 | Chorismate mutase (aroQ) | 186 | + | Periplasmic |  | + |
| ACICU_02544 | Superoxide dismutase | 209 | - | Periplasmic | + |  |
| AB57_0283 | Beta-lactamase TEM | 287 | + | Periplasmic |  | + |

^a^Secretome
